# When Feedback Matters: An Active-Inference Model of the Speaking-over-Writing Advantage in Production-Modality Retrieval

**DOI:** 10.3390/bs16101853

**Published:** 2026-10-09

**Authors:** Roberto Limongi, Oluwagbemisola Comfort Oguntoye, Angelica Maria Silva

**Affiliations:** 1 Department of Psychology, Faculty of Science, Brandon University, 270—18th Street, Brandon, MB R7A 6A9, Canada; oguntooc60@brandonu.ca; 2 Department of Francophone Studies and Languages, Faculty of Arts, Brandon University, 270—18th Street, Brandon, MB R7A 6A9, Canada; silvaa@brandonu.ca

**Keywords:** active inference, free-energy principle, Markov decision process

## Abstract

People often recognize spoken words more accurately and more quickly than written words. From the perspective of active inference under the free-energy principle, the present study develops a formal account of this speaking-over-writing advantage in production-modality retrieval. We modeled retrieval under spoken and written production-modality states, together with the selection of an early or late response policy according to its expected sensory consequences. Sixteen university students spoke or wrote words presented on a computer screen and later judged the modality in which each word had been produced. Responses were followed by explicit visual feedback indicating whether each judgment was correct or incorrect, thereby providing an observable sensory consequence of the recognition response. Response times were implemented as early and late policies within a partially observable Markov decision process (POMDP). The model included modality-specific likelihood parameters linking response timing to feedback outcomes. Spoken words were identified more quickly and accurately than written words, and early responding was more strongly associated with correct feedback under the speaking state than under the writing state. The POMDP was compared with a simpler Variational-Laplace model with a logistic observation function. Bayesian model selection favored the POMDP, indicating that the POMDP provided a better balance of fit and complexity. These findings show how production modality, response timing, and expected sensory consequences can be represented within a common generative framework. The framework provides a basis for future extensions that model uncertainty about production modality, preserve continuous response timing, incorporate learning across trials, and compare active-inference accounts with alternative models of recognition memory.

## 1. Introduction

Imagine meeting someone for the first time and learning their name, Ana. During the encounter, the person says the name aloud, creating a production episode that includes multiple contextual features (CF), such as hearing their own voice, producing a particular pronunciation, executing the corresponding articulatory movements, and experiencing the social context in which the name was spoken. What appears to be a simple social convention illustrates a broader principle in cognitive psychology: memory is shaped not only by the item to be remembered, but also by CF generated during encoding (1, 2), including the fact that the item was spoken aloud.

One week later, when meeting the same person again, the earlier speaking episode may be partially reinstated by a cue such as, “You said my name with your unique accent.” This contextual reinstatement can support a response policy: the person may respond relatively early—“Oh yes! How are you, Anna?”—or hesitate and respond later. Importantly, the response is selected before its consequences are observed. An early response may be expected to produce confirming feedback if the retrieved name is correct, but it may instead produce corrective feedback, such as “No, it’s Ana!” if the retrieval is inaccurate. The example therefore distinguishes the expected sensory consequences that contribute prospectively to policy evaluation from the actual feedback observed after the response.

**Figure 1:**
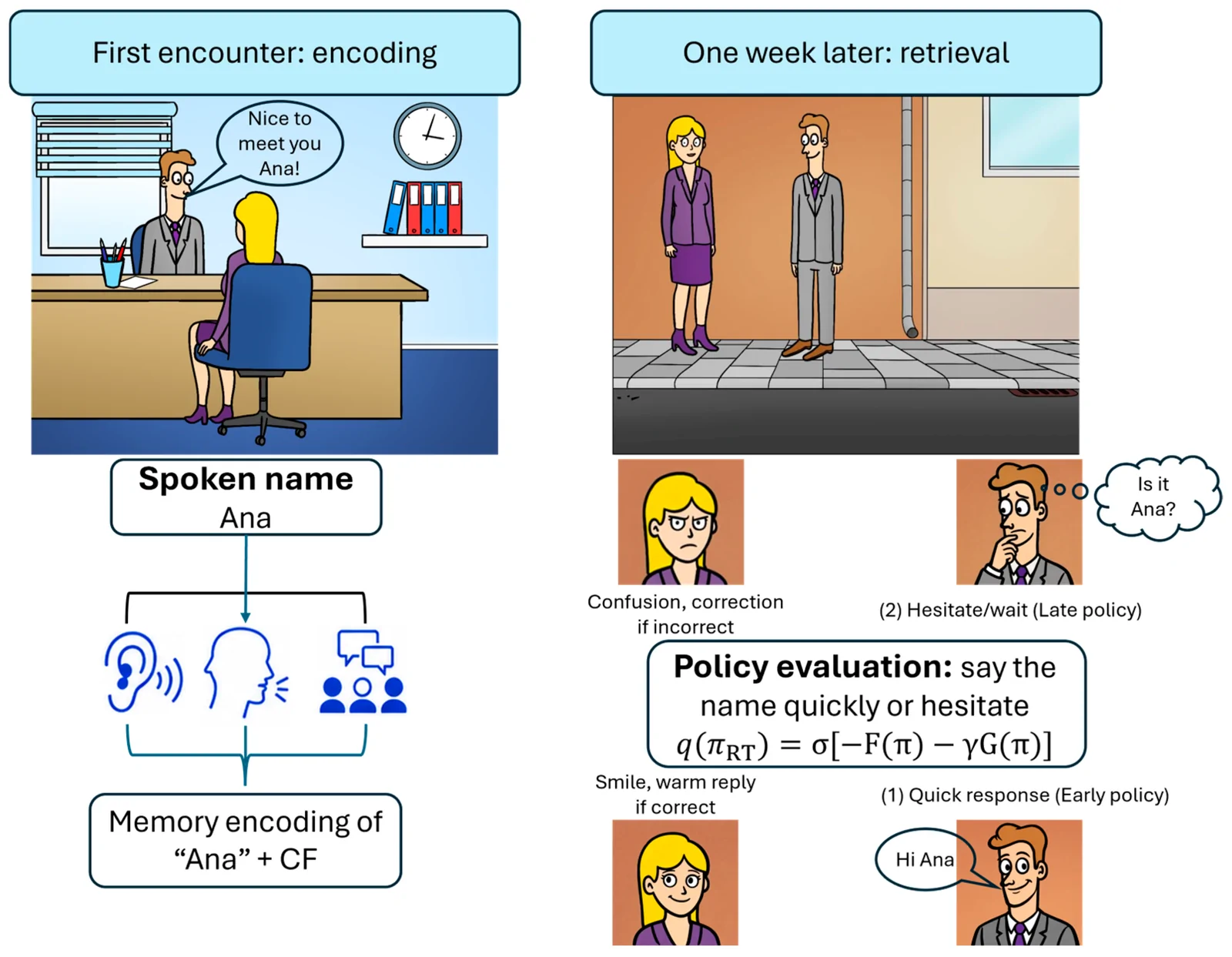
Everyday example of production-modality retrieval as policy evaluation. The equation on the right represents the posterior probability of selecting a response policy, as described in Section 1.1.2 and Section 1.1.3.

Although the person may strongly believe that the retrieved name is correct, its correctness is not directly observable. It must instead be inferred from sensory observations. In this example, the relevant observation is the other person’s response. A smile provides sensory evidence that the person, acting as an active-inference agent (Friston et al., 2025), can use to update their belief that the retrieved name was correct. Conversely, confusion or correction provides evidence that the retrieval was inaccurate. Approving feedback therefore matters because it serves as an observable consequence of an otherwise hidden state: whether the retrieved name is correct (Figure 1).

The introductory real-life example reflects the well-established finding that word-recognition accuracy is higher for words that participants produce aloud than for words they read silently (Bodner et al., 2016; Caplan & Guitard, 2024; Cyr et al., 2022; Fawcett et al., 2025; Forrin et al., 2012; Gionet et al., 2022; Icht et al., 2014; Jamieson et al., 2016; Kelly et al., 2022, 2024; MacLeod, 2011; MacLeod & Bodner, 2017; MacLeod et al., 2010; Mama, 2024; Mama et al., 2018; Mama & Icht, 2019; Ozubko & Macleod, 2010; Saint-Aubin et al., 2021; Wakeham-Lewis et al., 2022). Most experiments examining this phenomenon use a two-stage task. During the production stage, participants either read words aloud or read them silently. During the subsequent retrieval stage, previously presented words are intermixed with novel lures, and participants judge whether each item appeared during the production stage.

Enhanced recognition is not limited to words spoken aloud. Writing a word can also improve its subsequent recognition (Forrin et al., 2012). Crucially, this benefit is typically weaker than that associated with speaking the word aloud (Bodner et al., 2016; Forrin et al., 2012; Kelly et al., 2024; MacLeod & Bodner, 2017). This pattern gives rise to a speaking-over-writing advantage, whereby spoken words are generally recognized more accurately than written words.

Contemporary theoretical neurobiology, particularly active-inference accounts, proposes that cognition and action are organized partly around their anticipated sensory consequences (Friston, 2009, 2010; Friston et al., 2016a, 2025). In this view, an agent does not select an action solely on the basis of currently available evidence, but also according to what the action is expected to make observable. In the introductory example, retrieving the name “Ana” is therefore not only a matter of reinstating information about the earlier production episode. It is also embedded in the evaluation of a possible action—saying the name—and of the sensory consequences expected to follow from that action, such as a smile, a warm response, confusion, or correction.

The present account applies this active-inference assumption specifically to the retrieval of production-modality traces. When a participant infers that a word was previously spoken or written, that inference provides evidence about whether the item was encountered before. However, the functional significance of the trace also depends on how it informs the selection of a recognition response expected to produce a preferred observation, such as feedback confirming that the judgment was correct. Within active inference, this aspect of policy evaluation is captured by pragmatic value: policies are favored to the extent that they are expected to bring about preferred observations and avoid non-preferred observations. Retrieval is therefore treated not as an isolated reconstruction of the encoding episode, but as part of an action-oriented inferential process in which beliefs about prior production and beliefs about future sensory consequences jointly contribute to recognition-policy evaluation.

The above general account can be applied to provide a preliminary formalization of the speaking-over-writing advantage in word retrieval. Specifically, spoken and written production-modality traces may differ in the reliability or precision with which they support this process. A spoken trace may provide more distinctive or diagnostically informative evidence because it includes auditory and articulatory information, whereas a written trace may rely more heavily on visuomotor and orthographic information. A more precise spoken trace would support stronger beliefs about the earlier production modality, and consequently, greater confidence in selecting a recognition policy expected to yield confirming sensory feedback. From this perspective, the speaking-over-writing advantage can be characterized as a difference in the precision with which spoken and written production-modality traces inform recognition-policy selection in relation to the sensory consequences expected from responding.

The active-inference account therefore predicts that modality-specific likelihood mappings will shape the expected pragmatic value of alternative response-timing policies. The condition-specific likelihood parameters specify how strongly early and late response states are expected to generate correct or incorrect feedback under speaking and writing. Because correct feedback is preferred and incorrect feedback is avoided, these likelihood mappings determine the expected pragmatic value of the available response-timing policies. A speaking context in which early responding is more strongly associated with correct feedback should therefore confer greater pragmatic value on the early-response policy, whereas a writing context in which this mapping is weaker should shift relative pragmatic value toward later responding.

This modality-dependent pragmatic asymmetry is latent. It is not directly observed in response time or accuracy but is implied by the generative model and is expected to bias prospective posterior beliefs over response-timing policies, and subsequently, action probabilities. The specific theoretical contribution of the current model therefore lies in formalizing a path from modality-specific likelihood mappings to latent pragmatic valuation, prospective policy inference, and overt response timing. Through this inferential sequence, the model can also reproduce the observed condition-specific distribution of early and late responses.

### 1.1. The Current Work

The present paper pursues a specific theoretical objective: to develop a formal, yet preliminary, active-inference model, implemented as a partially observable Markov decision process (POMDP), of the speaking-over-writing advantage in production-modality retrieval. The model characterizes how modality-specific likelihood mappings between response timing and feedback generate differences in the expected pragmatic value of alternative response-timing policies and how these differences are expressed through prospective posterior beliefs over policies and subsequent action probabilities. In this framework, production-modality CF provide the contextual state under which the expected sensory consequences of early and late responding are evaluated, allowing the model to formalize a path from modality-specific likelihood precision to pragmatic policy evaluation and overt response timing.

This modeling work is intentionally narrow. It does not aim to explain every way in which CF enhance memory retrieval. Instead, it formalizes one specific component of this process: how spoken and written production-modality traces, as elements within a broader set of CF, guide retrieval within an active-inference framework (Auksztulewicz & Friston, 2015; Feldman & Friston, 2010; Parr & Friston, 2017, 2019; Parr et al., 2022; Smith et al., 2022). In this framework, explicit feedback provides the sensory evidence through which beliefs about the appropriateness of the selected response can subsequently be updated.

The remainder of the manuscript is organized as follows. First, we briefly introduce the experimental task we used to instantiate the POMDP. Second, we describe the model specification and its mathematical formulation. Third, we report descriptive statistical analyses confirming the speaking-over-writing advantage elicited by the task, followed by the POMDP parameter estimates obtained by fitting the model to the collected data.

#### 1.1.1. Word-Production and Retrieval Task That Instantiates the POMDP

**Figure 2:**
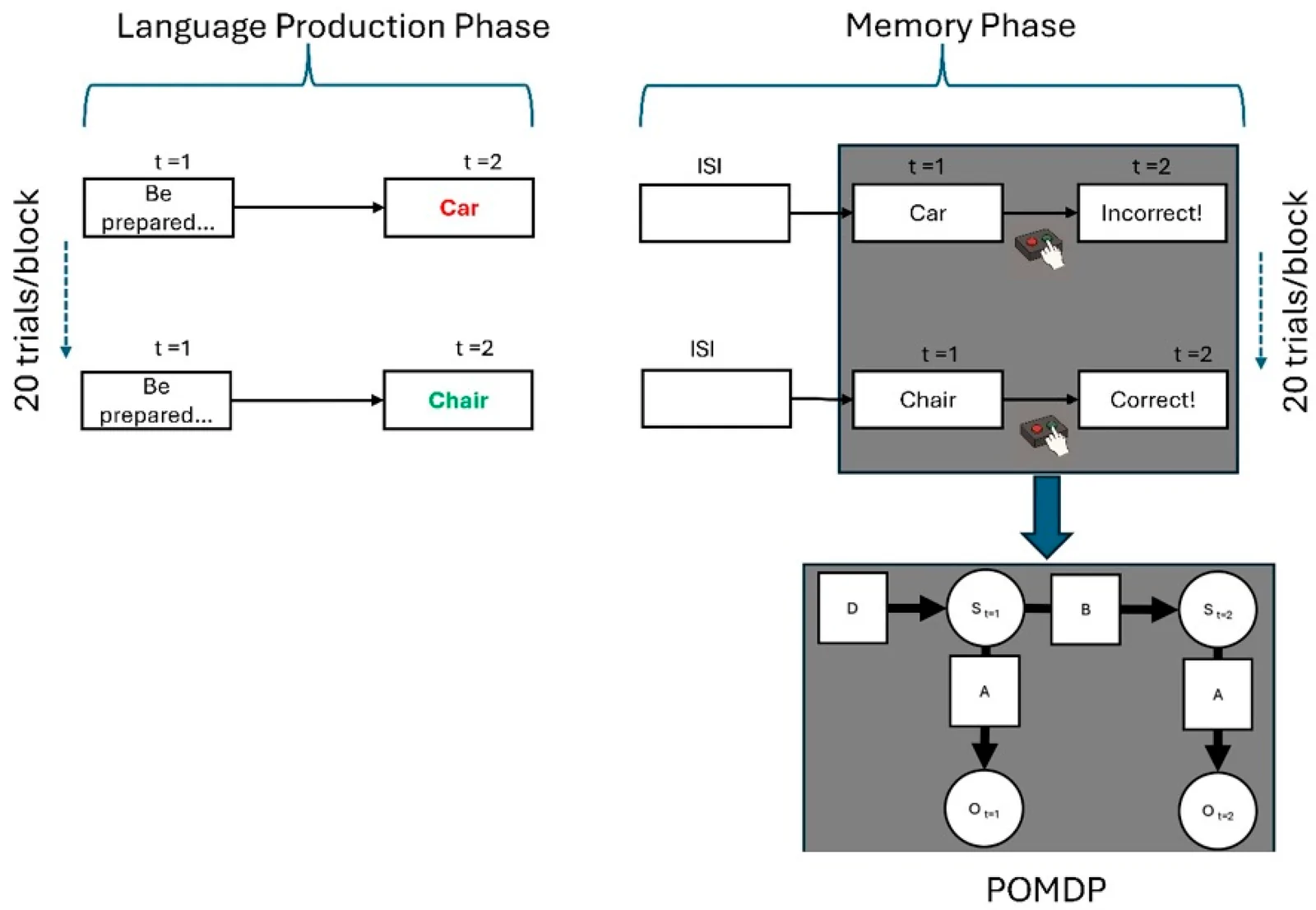
Experimental task block. Participants performed 9 blocks of 20 trials in two phases: language production phase and memory phase. As shown in the production phase, the language modality was cued by the word color (speaking in red, writing in green) counterbalanced across participants. In the memory phase, participants responded by pressing relevant keys whether the word was spoken or written (colored buttons do not represent the actual response keys which were “S” for speaking and “W” for writing). Participants observed feedback at the time of response. A partially observable Markov decision process model (POMDP) instantiates the two timesteps corresponding to the memory phase. In the model (see text for details), the response state transition (from no response at timestep one to early or late response state at timestep 2) is dictated by the B matrix. The A matrix expresses the likelihood of observing no-feedback, incorrect, or correct feedback at each timestep.

The task (see the Materials and Methods section for details) comprised production and retrieval stages (Figure 2). During the production stage, participants spoke or wrote blocks of words presented on a computer screen. During the subsequent retrieval stage, previously produced words were presented again, and participants indicated whether each word had originally been spoken or written.

After each judgment, participants received explicit visual feedback indicating whether their response was correct or incorrect. This feedback constituted an observable sensory consequence of the recognition response. Correct feedback represented a preferred outcome, whereas incorrect feedback represented an outcome to be avoided. The task therefore allowed recognition behavior to be modeled not only in terms of beliefs about the earlier production modality, but also in terms of the sensory outcomes that different responses were expected to generate.

A further feature of the task was that participants were free to determine when to respond after the retrieval prompt appeared. Response time therefore indexed when participants acted on their current belief about the production modality. In the discrete POMDP, this temporal aspect of behavior had to be represented as a choice between a finite set of response-timing policies. The theoretical rationale for this representation follows from the distinction in active-inference formulations between continuous-state/action and discrete-state/action models (Da Costa et al., 2020; Parr et al., 2022; Silva et al., 2026; Skelton et al., 2026; Smith et al., 2022). Continuous-time formulations are suited to modeling continuously evolving states and movements, whereas discrete-time formulations represent inference over a finite set of hidden states, observations, actions, and policies. In the present model, the theoretically relevant quantity was not the continuous motor execution of the response itself but the policy governing when participants committed to and expressed their belief that a word had previously been spoken or written.

To instantiate these alternative temporal policies, response times were classified separately for each participant after collapsing across the speaking and writing conditions. Each participant’s median response time served as the threshold: responses equal to or below the median were coded as enactments of the early-response policy, whereas responses above the median were coded as enactments of the late-response policy. These policies represented acting relatively early on the current belief about production modality or delaying the response before committing to that belief. The participant-specific threshold expressed policy timing relative to each participant’s own response distribution rather than imposing a common absolute cutoff across participants.

A continuous-action formulation would instead be more appropriate for modeling the evolving motor dynamics through which a selected policy is executed, such as hand trajectories, keypress dynamics, speech articulation, or other continuously unfolding movements. Note, therefore, that the early–late classification was introduced to define the two temporal policies available to the agent within the discrete POMDP rather than merely to simplify a continuous response-time measure.

In summary, the memory phase of the task thus involved two related inferential components. First, participants formed a belief about whether the probe word had previously been spoken or written. Second, they selected when to act on that belief, with early and late responding represented as alternative policies associated with different expected feedback outcomes. Recognition accuracy and response timing were therefore treated jointly as observable expressions of the latent decision process underlying retrieval (Ratcliff, 1978). Within this framework, the central computational question is whether the speaking-over-writing advantage can be characterized by differences in the precision of the mapping from production-modality and response-timing states to feedback outcomes. Specifically, we examine whether early and late response policies differ in how reliably they are associated with correct feedback under spoken and written production-modality states. If spoken production supports a more precise retrieval trace than written production, an early response under the spoken state should be more strongly associated with correct feedback than an early response under the written state.

The following section describes the POMDP architecture through which production-modality states, response-timing policies, feedback observations, and their likelihood mappings are formally represented.

#### 1.1.2. Partially Observable Markov Decision Process

In active inference, policy selection is guided by expected free energy, which evaluates policies according to the outcomes they are expected to generate. Expected free energy includes a pragmatic component, related to attaining preferred outcomes, and an epistemic component, related to reducing uncertainty. In the present model, correct feedback constitutes a preferred outcome, whereas incorrect feedback constitutes an outcome to be avoided. Feedback also provides sensory evidence about the response generated under the current production-modality state. Participants are therefore represented as active-inference agents who select response-timing policies according to the feedback outcomes that those policies are expected to produce when retrieving words that were previously spoken or written.

**Figure 3:**
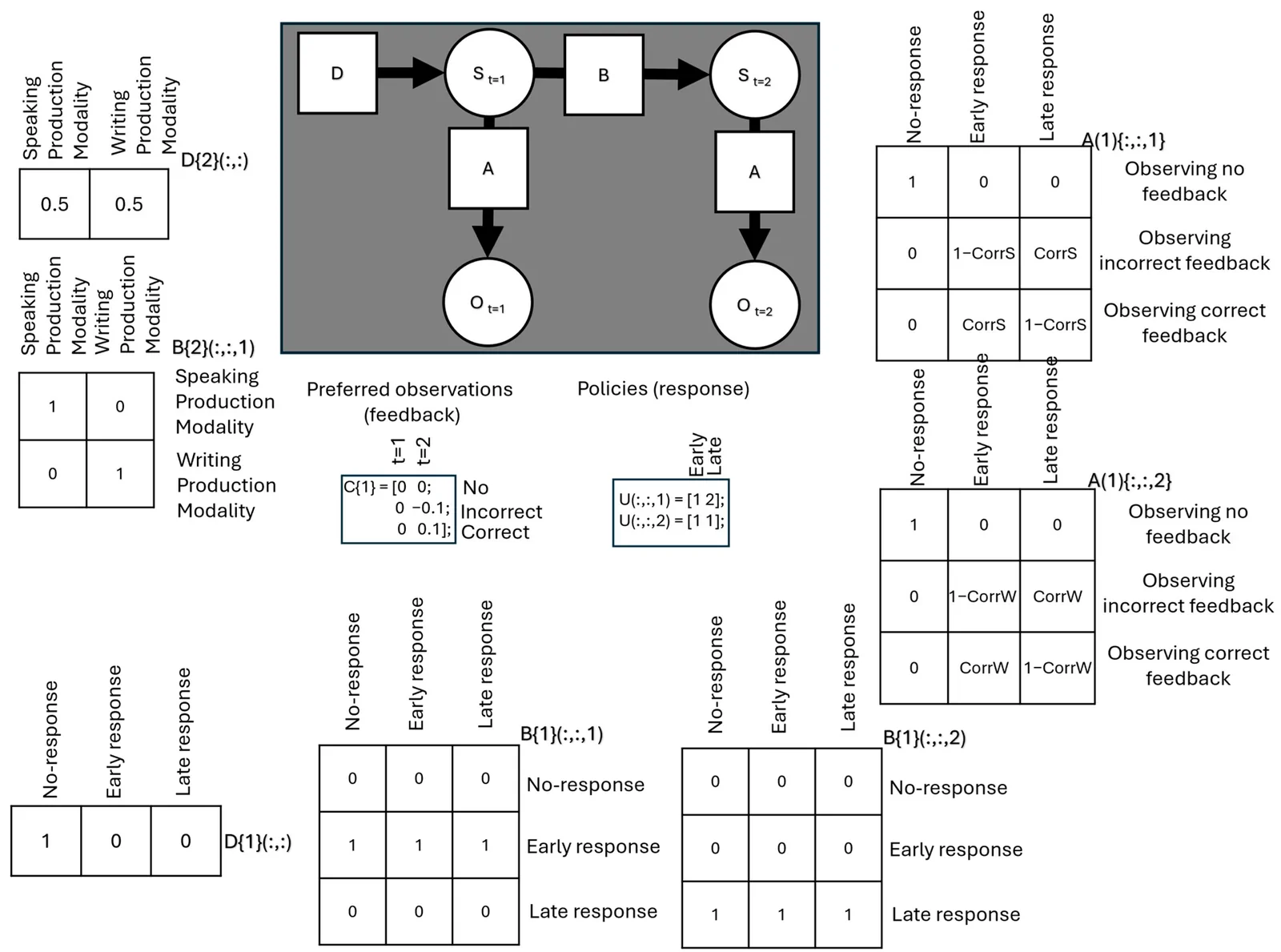
Factor graph of the POMDP model. CorrS—Correct-speaking parameter, CorrW—Correct-writing parameter. D matrix represents beliefs at the beginning of a trial. A matrix represents the state-outcome mappings. The B matrix represents Markovian transitions (policy-dependent state-transition probabilities). The C matrix encodes preferred observations (note that at t = 2 correct feedback is slightly preferred to incorrect feedback).

We formalized this process using a two-timestep POMDP model (Parr et al., 2022; Smith et al., 2022). The model represents the participant as an agent who selects a response-timing policy and subsequently updates beliefs after observing feedback. It specifies the hidden states, observable outcomes, likelihood mappings, preferences, policies, and state transitions required to represent this process. The production-modality state corresponds to the modality in which the probe word was encoded: speaking or writing. The agent selects either an early-response or a late-response policy and then observes correct or incorrect feedback. The central computational question is whether the mapping between response timing and feedback differs across the speaking and writing production-modality states. Figure 3 presents the POMDP specification as a factor graph.

The model contains two hidden-state factors. The first factor represents response state and comprises three states: no response, early response, and late response. This factor is action-dependent because the selected policy determines the transition from the no-response state at the first timestep to either the early-response or late-response state at the second timestep. These policy-dependent transitions are encoded in the B{1}(:,:,1) matrix. The second hidden-state factor represents production modality and comprises two states: speaking and writing. This factor indicates the modality in which the current probe word was originally produced. In the present implementation, the production-modality state is determined by the trial structure and remains stable across the two timesteps. Its transitions are encoded in the B{1}(:,:,2) matrix.

The model includes one outcome modality, represented by the A{1}(:,:,1) matrix, with three possible observations: no feedback, incorrect feedback, and correct feedback. Feedback is generated by the joint configuration of the response state and the production-modality state. At the first timestep, the agent occupies the no-response state and observes no feedback, regardless of whether the production-modality state is speaking or writing. At the second timestep, the agent occupies either the early-response or late-response state and observes correct or incorrect feedback.

The agent’s problem is therefore to select the response-timing policy expected to generate the preferred feedback outcome under the current production-modality state. Under the speaking state, an early response generates correct feedback with probability (CorrS), whereas a late response generates correct feedback with probability (1-CorrS). Under the writing state, an early response generates correct feedback with probability (CorrW), whereas a late response generates correct feedback with probability (1-CorrW).

The parameters CorrS and CorrW quantify the precision of the likelihood mapping from the joint configuration of production modality and response state to feedback. More specifically, they determine how reliably early, relative to late response state predicts correct feedback under the speaking and writing states. The speaking-over-writing advantage is therefore represented as a condition-specific difference in these likelihood parameters. If speaking supports earlier and more confident retrieval than writing, the early-response state should map more strongly onto correct feedback in the speaking state than in the writing state. Formally, this prediction corresponds to CorrS>CorrW.

Preferences over feedback observations are encoded in the C{1} matrix. No-feedback observations are assigned a neutral preference, incorrect feedback is avoided at the second timestep, and correct feedback is preferred at the second timestep. The two possible response timings are encoded as shallow policies in the (U) matrix, with one policy producing an early response and the other producing a late response.

The model additionally specifies parameters governing policy selection and action execution. The inverse policy-precision parameter is fixed at β = 1, allowing expected differences among policies to exert a relatively strong influence on policy selection. The action-precision parameter is fixed at α = 16, producing relatively consistent execution of the selected response policy. The fixed values were chosen to provide a weakly informative reference parameterization rather than to optimize fit. With β = 1, the model retained a standard (SPM-default) expected-free-energy contribution to policy selection whereas with α = 16, the model produced relatively precise but still stochastic action selection. Finally, with C = ±0.1, the model encoded only a modest preference for correct over incorrect feedback so that behavior would not be dominated by strong outcome preferences.

#### 1.1.3. Formal Decomposition of Policy Belief Updating

We next decomposed the *prospective posterior belief over response-timing policies*, $q\left(\pi_{RT}\right)$, in terms of variational free energy and precision-weighted expected free energy. Because the agent expects feedback at the second timestep, response timing is selected by sampling from a posterior distribution over policies after the no-feedback observation at the first timestep. This distribution is expressed as:

$$q\left(\pi_{RT}\right)=\sigma \left[-F\left(\pi \right)-\gamma G\left(\pi \right)\right],$$

Here, $q(\pi_{RT})$ denotes the prospectively updated posterior probability of selecting response-timing policy $\pi_{RT}$. The term $F\left(\pi_{RT}\right)$ denotes the policy-specific variational free energy associated with observations already available. The term $G(\pi_{RT})$ denotes the expected free energy of future feedback observations under that policy. The parameter γ  denotes policy precision and controls the extent to which differences in expected free energy influence policy selection.

In the SPM implementation, this prospective belief updating over candidate response-timing policies is expressed in (MDP.R), the posterior distribution over policies. These posterior policy beliefs are subsequently mapped onto posterior action probabilities (MDP.P), which determine the probability of selecting the early-or-late response action. Thus, the likelihood parameters influence behavior indirectly by shaping expected policy outcomes, which update beliefs over policies, and in turn, action probabilities.

Accordingly,$\;F\left(\pi_{RT}\right)$ evaluates how well a policy explains observations that have already occurred, whereas $G\left(\pi_{RT}\right)\;$ evaluates the expected pragmatic and epistemic value of observations that may occur in the future. Policies with lower variational free energy and expected free energy are assigned higher posterior probability.

In the present two-timestep model, before correct or incorrect feedback is observed, $F\left(\pi_{RT}\right)\;$ is determined primarily by the no-feedback observation and the production-modality state. Because both the early- and late-response policies are associated with no feedback at the first timestep, variational free energy provides little basis for distinguishing between them at that point. Initial policy selection is therefore driven mainly by expected free energy. Specifically, the agent evaluates whether the state reached under an early- or late-response policy is expected, given the current production-modality state, to generate the preferred observation of correct feedback at the second timestep.

#### 1.1.4. Pragmatic and Epistemic Decomposition of Expected Free Energy

For a response-timing policy, π, the expected free energy may be written as:

$$G(\pi )=E_{q(o_{\tau },s_{\tau }|\pi )}[\ln\;q(s_{\tau }|\pi )-\ln\;p(o_{\tau },s_{\tau }|\pi )].$$

Expected free energy can be decomposed into pragmatic and epistemic components. The pragmatic component evaluates whether a policy is expected to generate preferred observations, whereas the epistemic component evaluates whether it is expected to reduce uncertainty about hidden states. In the present model, the pragmatic value of a policy depends on its probability of generating correct rather than incorrect feedback.

$$G\left(\pi \right)=\underset{\mathrm{Epistemic}\;\mathrm{term}}{E_{q(o_{\tau },s_{\tau }|\pi )}[\ln\;q\left(s_{\tau }|\pi \right)-\ln\;q\left(s_{\tau }|o,\pi \right)]}-\underset{\mathrm{pragmatic}\;\mathrm{term}}{E_{q(o_{\tau },|\pi )}\left[\ln\;p(o|C)\right]}.$$

Feedback can, in principle, have epistemic value insofar as it reduces uncertainty about hidden states. In the present implementation, however, the epistemic contribution is limited. Production modality is *fixed* by the trial structure and therefore does not need to be inferred from feedback, while the response-timing state is strongly constrained by the selected policy and state-transition structure. Consequently, the principal policy-discriminating contribution of expected free energy in the present model is pragmatic: alternative response-timing policies differ primarily in the preferred feedback outcomes they are expected to generate.

To illustrate the role of the likelihood parameters, consider a speaking context in which the early-response policy moves the agent from the start state into the early-response state. Feedback is not generated directly by the policy itself. Rather, the expected feedback depends on the joint configuration of the response-timing state and the production-modality state through the likelihood mapping. Thus, under the speaking state, suppose that occupying the early-response state is associated with correct feedback with a probability of 0.60 and incorrect feedback with a probability of 0.40.

The pragmatic value ($V_{prag})$ of a policy $\left(\pi \right)$ can be expressed as the expected preference over the observations that are likely to follow from the state reached under that policy:

$$V_{prag}\left(\pi \right)=\sum_{o}P\left(o|\pi \right)C\left(o\right).$$

In the present model, the relevant outcome probabilities are induced by the state reached under the policy and the current production-modality state. More explicitly,

$$P\left(o|\pi \right)=P\left(o,|s_{\mathrm{timing}}\left(\pi \right)s_{\mathrm{modality}}\right),$$

so that

$$V_{prag}\left(\pi \right)=P\left(correct,|s_{\mathrm{timing}}\left(\pi \right)s_{\mathrm{modality}}\right)C_{correct}+P\left(incorrect,|s_{\mathrm{timing}}\left(\pi \right)s_{\mathrm{modality}}\right)C_{incorrect}.$$

If correct feedback has preference +0.1 and incorrect feedback has preference −0.1, then for the early-response policy under speaking:

$$V_{prag}\left(\pi_{early}\right)=\left(0.60\right)\left(0.1\right)+\left(0.40\right)\left(-0.1\right)=0.02.$$

The late-response policy instead moves the agent into the late-response state. Under the complementary likelihood mapping, suppose that the joint configuration of the late-response state and the speaking state is associated with correct feedback with a probability of 0.40 and incorrect feedback with a probability of 0.60. Its pragmatic value is therefore:

$$V_{prag}\left(\pi_{late}\right)=\left(0.40\right)\left(0.1\right)+\left(0.60\right)\left(-0.1\right)=-0.02.$$

The likelihood mapping therefore creates a latent pragmatic advantage of:

$$0.02-\left(-0.02\right)=0.04$$

for the early-response policy under speaking. Under a writing state with the opposite likelihood mapping, the direction of this pragmatic advantage reverses, so that the late-response policy acquires greater pragmatic value.

In this way, the likelihood parameters do not function as descriptive end points. They specify how the joint configuration of response-timing state and production-modality state is expected to generate correct or incorrect feedback. Those expected observations, weighted by their preferences, determine the pragmatic value of the candidate policies and thereby contribute to prospective policy inference.

#### 1.1.5. Variational Free Energy and Variational Message Passing During Belief Updating

Although variational free energy has limited policy-discriminating value at the first timestep, it becomes important after feedback is observed. Once the agent has selected a response-timing policy and received correct or incorrect feedback, it must update its beliefs to account for that observation. This updating is implemented through marginal message passing (Parr et al., 2019; Smith et al., 2022), with variational free energy supplying the objective function:

$$F(\pi )=D_{KL}[q(s)∥P(s|o)]-\ln\;P(o).$$

Minimizing variational free energy reduces the divergence between the approximate posterior, q(s), and the Bayesian posterior, P(s∣o). In the present model, this means that after feedback is observed, beliefs about the response state are updated so that they better explain the feedback under the current production-modality state.

In summary, $G\left(\pi \right)$ evaluates response-timing policies prospectively, before feedback is observed, according to their expected pragmatic and epistemic consequences. In contrast, $F\left(\pi \right)\;$ evaluates how well a policy and its associated hidden states explain observations that have already occurred. At the first timestep, both policies predict no feedback, and $F\left(\pi \right)$ therefore provides little discrimination between them. At the second timestep, after correct or incorrect feedback is observed, variational free energy supports retrospective belief updating by evaluating how well the inferred hidden-state configuration accounts for the observed outcome under the likelihood mapping.

## 2. Materials and Methods

### 2.1. Participants

Under laboratory conditions, 16 undergraduate students (6 biological males and 10 biological females; M age = 22.5, SD = 6.22) from Brandon University were recruited via campus advertisements. Sample size estimation was based on sequential analysis and optional stopping (Rouder, 2014; Tendeiro et al., 2022). Participation was voluntary, and all individuals provided written informed consent prior to the initiation of the experiment. All participants received a $50 gift card as compensation for their time. The study was approved by the Brandon University Research Ethics Committee.

#### Optional Stopping for Sample Size Definition

After data had been collected from the first 10 participants, we fitted Bayesian ANCOVA models to the response-time and accuracy data, with condition as a fixed effect, participant as a random effect, and word frequency as a covariate. Before data collection, we specified an optional-stopping rule according to which the first Bayesian ANCOVA would be conducted after 10 participants and then repeated after each additional participant until the evidence reached BF_10_ > 10, conventionally interpreted as strong evidence (Jeffreys, 1961) for higher accuracy and faster response times in the speaking condition than in the writing condition. This stopping rule was defined in advance, although it was not formally preregistered with the journal.

**Figure 4:**
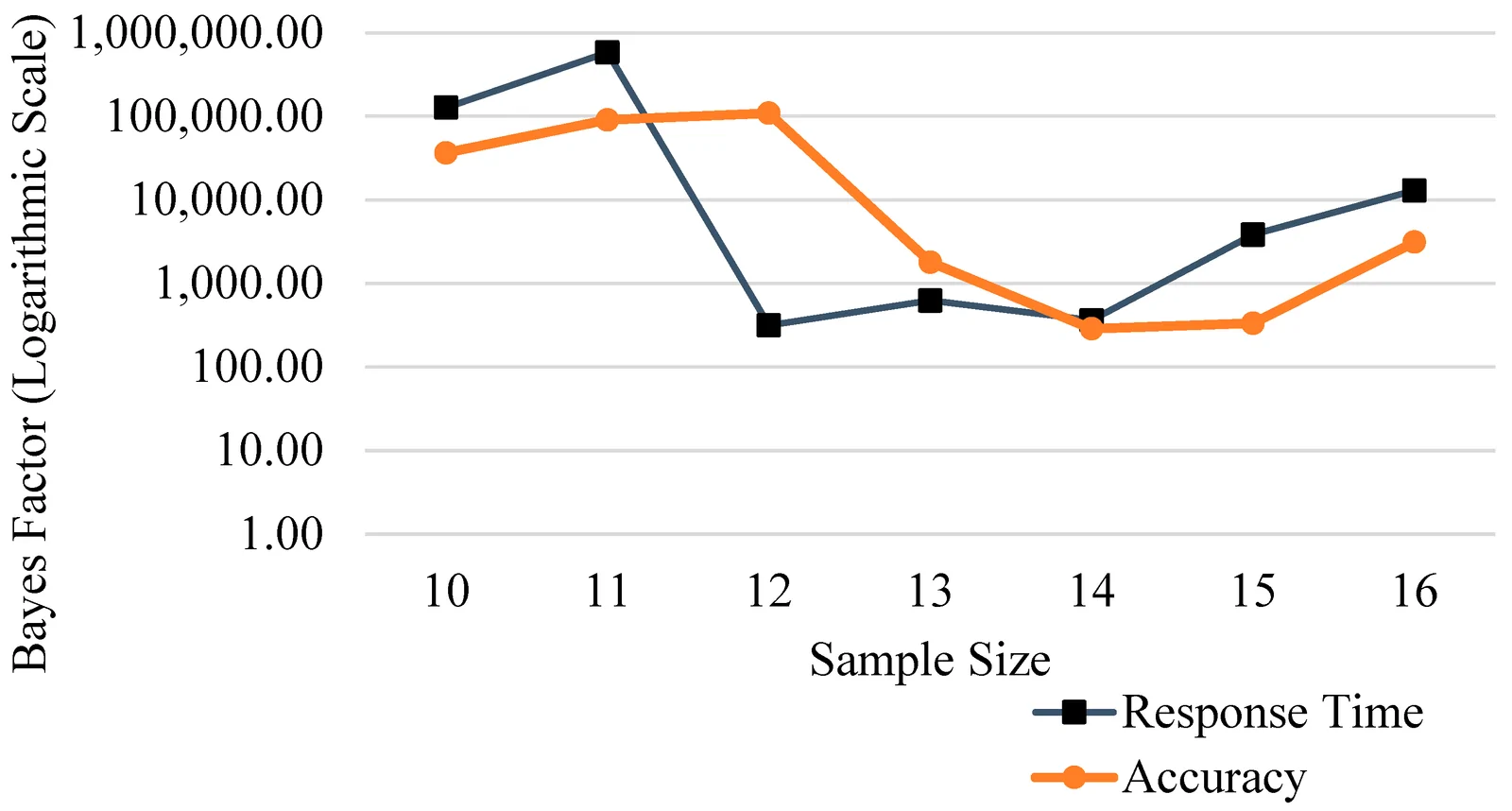
Robustness check of sample size definition using optional stopping.

The evidential threshold had already been exceeded when the first analysis was conducted at N = 10, such that data collection could have ended at that point according to the predefined rule. We nevertheless continued data collection to examine whether the evidential conclusion remained stable at BF_10_ > 10 as additional participants were added. Specifically, we increased the sample by 50%, from N = 10 to N = 15, recomputing and recording the Bayes factor after each additional participant. After confirming that the Bayes factor remained above the stopping threshold throughout this sequence, we recruited one additional participant to preserve the intended counterbalancing, yielding a final sample of N = 16. Figure 4 reports the sequential Bayes factors. This procedure therefore combined a predefined Bayesian optional-stopping rule with an additional robustness check.

### 2.2. Stimuli

Our stimuli consisted of an experimental word list of 180 nouns with the highest academic frequency selected from the Strathy Corpus of Canadian English (Strathy Language Unit, 2024). For each participant, the order of the 180 words was randomized, and the list was split into two sublists (one per condition) of 90 words each. This randomized order was used to assign each word, without replacement, to a language condition (writing or speaking). Nine block lists of 20 words each were then created, with words randomly drawn from the speaking and writing sublists (10 words per condition).

### 2.3. Procedure and Software

Participants were seated at a computer and wore noise-cancelling headphones throughout the experiment. Each participant completed nine blocks, each consisting of a language production phase followed by a memory phase (Figure 2). After completing each block, participants took a break and were free to decide when to begin the next block by pressing the space bar. The experiment began with four familiarization trials (two per condition). In the production phase of each block, participants were presented with 20 words for 5 s each, displayed either in red ink (speaking condition) or green ink (writing condition)—counterbalanced across participants. The color of the word cued participants to either speak (speaking condition) or type (type-writing condition, hereafter writing condition) the word. Each word presentation was preceded by a “be prepared” slide displayed for 4.5 s. After completing the language production phase, participants performed the memory phase using the same words presented during the production phase. A memory trial began with a blank screen (5 s) serving as a fixed inter stimulus interval, followed by a probe word (5 s, unless a response was recorded) and feedback screen (“correct”, “incorrect”, or “no response recorded”, 5 s). Probe words were displayed in black ink on a white background. The task was built using PsychoPy, 2024.2.4 which recorded accuracy, response time, and word production. Accuracy of word production (i.e., whether the participant actually spoke or wrote the target word) was confirmed via PsychoPy output (for written registers) and spectrogram analysis (for spoken registers). For basic statistical analysis, we used JASP 0.19.2.0 (JASP Team, 2024). For modeling, we used SPM12 (http://www.fil.ion.ucl.ac.uk/spm/, accessed on 18 July 2026) and modified scripts provided by Smith et al. (2022, 2020).

## 3. Results

### 3.1. Behavioral Results: Bayesian ANCOVA and Variational Laplace Logistic Analyses

We excluded trials in which feedback was “no response recorded”, indicating that participants did not provide either of the two allowed key presses, “S” or “W”, within the five-second response window. We applied this exclusion criterion equally across conditions. Table 1 shows the condition-wise mean response time and mean accuracy at the group level, indicating that participants responded faster and more accurately in the speaking condition than in the writing condition.

We fit Bayesian ANCOVA models to assess the support that the data provided for these differences (production-effect models) and to rule out the possible effect of word frequency (covariate models). The production-effect models included production modality as a fixed effect and participant as a random effect, whereas the covariate models additionally included word frequency as a covariate. Coefficient priors were set to JASP’s default values: r = 0.50 for fixed effects, r = 1 for random effects, and r = 0.354 for covariates. We compared the production-effect and covariate models relative to a reference null model. Table 2 shows the models’ parameter estimates and uncertainties.

For the effect on continuous response time, the production-effect model was more strongly supported than the covariate model (BF_10_ = 12,389 vs. BF_10_ = 5441). The posterior estimates indicated shorter response times for speaking than for writing. Similarly, for the effect on accuracy, the production-effect model was likewise better supported than the covariate model (BF_10_ = 3191 vs. BF_10_ = 293). Accuracy was higher in the speaking condition than in the writing condition.

To confirm that the production-modality difference in response time remained after applying the participant-specific median discretization strategy necessary for the main POMDP model, we additionally fit a Gaussian-error Variational Laplace (VL) (Zeidman et al., 2023) model with a logistic observation function to the discretized response times. Early responses were coded as 0 and late responses as 1. The design matrix included an intercept, production modality, and accuracy, with Speaking coded as 0 and Writing as 1. The logistic observation function constrained trial-wise predictions to the interval [0, 1], while residual prediction errors were evaluated under the Gaussian precision structure of the VL inversion. We fit one model per participant and used Parametric Empirical Bayes (PEB) (Friston et al., 2016b) at the group level. The resulting hierarchical estimate closely reproduced the earlier ANCOVA results (Table 2). Specifically, the exponentiated group-level PEB-coefficient posterior mean $e^{\beta =0.373}=1.45$ indicates that, holding accuracy constant, writing increased the odds of a late response relative to speaking by 45%. Crucially, the participant-specific variational free energies from this logistic VL model were subsequently compared with those of the POMDP using random-effects Bayesian model selection (Section 3.2.5).

### 3.2. Computational Model Results

#### 3.2.1. Parameter Recoverability Analysis

We evaluated parameter recoverability using synthetic datasets generated from known values of the two condition-specific likelihood parameters, CorrS and CorrW. Both parameters were varied independently from 0.10 to 0.90 in increments of 0.10, yielding 81 unique parameter combinations. For each combination, synthetic 90-trial sequences were generated separately for the speaking and writing conditions and then re-estimated using the same VL inversion procedure applied to the empirical data.

To ensure that recovery assessed the full generative architecture rather than simply refitting fixed observations, both feedback observations and response-timing actions were generated from the model. Recovery performance was quantified by correlating the generating and recovered parameter values across the full parameter grid. Bayesian Pearson correlations (Beta prior width = 1) showed that the generating and recovered values were highly associated for CorrS (r=0.964, $BF_{10}=4.06\times 10^{43}$) and CorrW  (r=0.958, $BF_{10}=1.77\times 10^{41}$). These results provide evidence that the inversion procedure recovered the generating parameter values across the tested parameter space and support the identifiability of the principal likelihood parameters under the present model architecture.

#### 3.2.2. Model Parameter Estimates

The subject-level condition-specific likelihood estimates (Table 3) show the predicted condition asymmetry. The mean speaking likelihood parameter was CorrS=0.532  (SD=0.040) whereas the mean writing likelihood parameter was CorrW=0.470  (SD=0.041). At the group level, PEB confirmed this difference: the posterior mean CorrS−CorrW contrast on the probability scale was 0.064. The 95% credible interval (CI) was [0.027, 0.102]), with $P\left(\Delta Corr>0\right)=0.99$.

As requested by one reviewer, we assessed whether a more flexible four-likelihood-parameter model provided a better account of the data than the specified two-likelihood-parameter model. Whereas the latter estimated one condition-specific likelihood parameter for speaking and one for writing, the four-parameter model estimated separate likelihood parameters for early and late responses within each condition, thereby relaxing the complementarity constraint imposed by the two-parameter model. We fitted the more flexible model to the data and compared the two models using random-effects Bayesian model selection. The comparison favored the more restrictive two-parameter model, with a protected exceedance probability of 0.99 and a Bayesian omnibus risk of <0.001.

We also examined the posterior covariance between the two condition-specific likelihood parameters to assess whether their estimates were statistically coupled during first-level inversion. For each participant, the posterior correlation between $Corr_{S}$ and $Corr_{W}\;$ was computed from the corresponding elements of the posterior covariance matrix, DCM.Cp. Under the planned median-based discretization, these posterior correlations were effectively zero across participants (mean $r\approx -7.2\times 10^{-10}$). This indicates that uncertainty in $Corr_{S}\;$ was essentially independent of uncertainty in $Corr_{W}$, and that the estimated group difference between the two parameters was not driven by a posterior trade-off between them.

To assess whether the difference in likelihood parameters depended on the particular median cutoff, we conducted a post hoc sensitivity analysis using participant-specific 25th-percentile (Q25) and 75th-percentile (Q75) response-time thresholds. We expected that altering the cutoff would change the overall proportion of trials classified as early. Indeed, at Q25, early responses comprised 29.2% of speaking trials and 20.9% of writing trials whereas at Q75, the corresponding proportions were 77.8% and 72.2%. Furthermore, the absolute likelihood estimates also shifted with the discretization threshold. At Q25, the mean speaking parameter was $Corr_{S}=0.366$ (SD=0.041) and the mean writing parameter was $Corr_{W}=0.303$ (SD=0.044). At Q75, $Corr_{S}=0.686$ (SD=0.036) and $Corr_{W}=0.642$ (SD=0.036).

Despite these shifts, the theoretically relevant ordering was preserved. The PEB posterior mean $Corr_{S}-Corr_{W}$ contrast was 0.067 at Q25 (95% CI [0.029, 0.105], $P\left[\Delta Corr>0\right]=0.99$) and 0.047 at Q75 (95% CI [0.009, 0.083], $P\left[\Delta Corr>0\right]=0.99$). Thus, the post hoc sensitivity analyses supported the robustness of the planned median-based result, indicating that the $Corr_{S}>Corr_{W}$ asymmetry was not specific to the median discretization criterion.

As an additional diagnostic step, we examined the posterior covariance structure under the Q25 and Q75 discretizations. The posterior correlations between $Corr_{S}$ and $Corr_{W}$ were again effectively zero across the participants, with mean posterior correlations of approximately $1.3\times 10^{-9}$ at Q25 and $-3.8\times 10^{-10}$ at Q75. Thus, the near-independence of the two parameter estimates was preserved across alternative discretization thresholds. This supports the interpretation that the robustness of the $Corr_{S}>Corr_{W}$ contrast was not an artifact of posterior dependence between the two likelihood parameters.

#### 3.2.3. POMDP Fixed-Modeling Choices Sensitivity Analysis

As indicated in the POMDP model specification (Section 1.1.2). Values of α=16,β=1,and C=+/−0.1 were fixed modeling choices. To determine whether the substantive inference depended on these choices, we conducted a full factorial sensitivity analysis in which $Corr_{S}$ and $Corr_{W}$ remained free parameters while C, α, and β were systematically varied. Specifically, we evaluated $C\in \left\{0.1,0.2,\ldots ,1.0\right\}$, $\alpha \in \left\{4,8,12,16,20,24,28,32\right\}$, and $\beta \in \left\{1,4,8,12\right\}$, yielding 320 model specifications fitted separately to each participant.

Across this model space, the substantive condition effect was stable: all 320 model specifications yielded a positive group-mean difference $Corr_{S}-Corr_{W}$. Bayesian paired-samples *t* tests were applied using this directional alternative hypothesis separately for each of the 320 C × α × β sensitivity specifications with a positive half-Cauchy prior on the standardized effect and scale r = 0.707. The directional inference was highly stable across the sensitivity space. Of the 320 models, 306 models (95.6%) yielded BF_+0_ > 3, indicating at least moderate evidence for the predicted directional effect, and 282 models (88.1%) yielded BF_+0_ > 10, indicating strong or stronger evidence. Only five specifications yielded BF_+0_ < 1, and none yielded BF_+0_ < 1/3. Taken together, these results indicate that the predicted CorrS > CorrW asymmetry is highly robust to variation in the fixed C, α, and β values.

#### 3.2.4. Posterior Predictive Check

**Figure 5:**
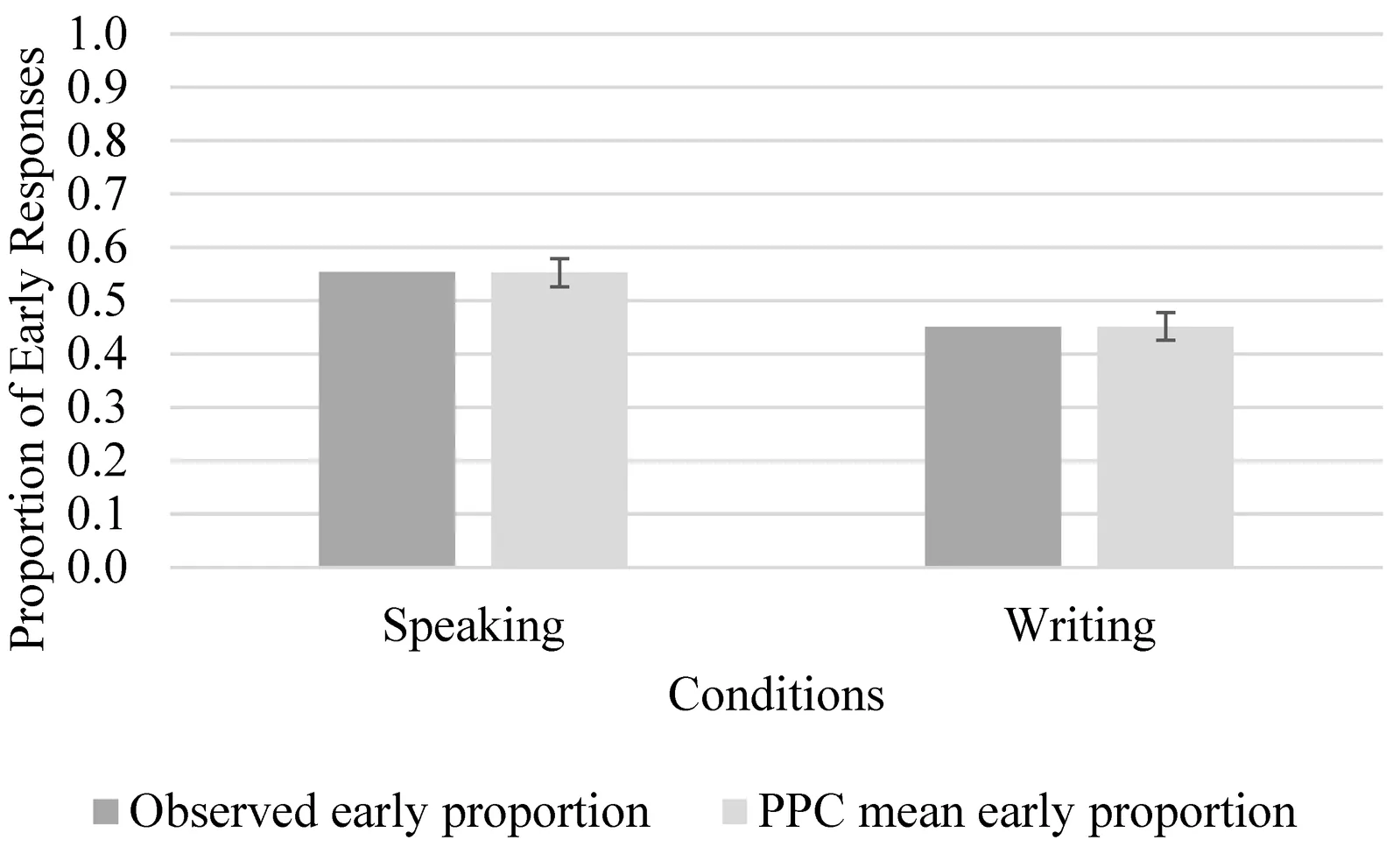
Posterior predictive check of early-response proportions by production condition. Bars show the observed proportion of early responses and the mean proportion generated across 1000 empirical-trial-matched posterior predictive replications. Error bars indicate the 95% posterior predictive interval, defined as the central 95% of simulated early-response proportions across replications. In both speaking and writing, the observed proportion fell within the corresponding posterior predictive interval.

Posterior predictive checks were conducted using 1000 empirical-trial-matched replicated datasets. For each participant, the fitted model was used to generate early-versus-late responses while preserving the participant-specific trial structure, language condition, and observed feedback sequence. Collapsing across feedback accuracy, the model closely reproduced the condition-specific distribution of response timing. Figure 5 depicts the group-level proportions of early responses by condition. We report the 95% posterior predictive interval (PPI), which represents the central 95% of early-response proportions generated across the 1000 replicated datasets. In speaking, 731 out of 1320 observed trials were classified as early (0.554), compared with a posterior predictive mean of 0.553 (95% PPI [0.527, 0.579]); correspondingly, the observed and predicted late-response proportions were 0.446 and 0.447. In writing, 598 out of 1327 observed trials were early (0.451) compared with a posterior predictive mean of 0.451 (95% PPI [0.426, 0.478]); the observed and predicted late-response proportions were therefore 0.549 and 0.549, respectively. The 95% posterior predictive interval represents the central 95% of early-response proportions generated across the 1000 replicated datasets. The observed early-response proportion fell within this interval in both conditions, indicating that the fitted model reproduced both the direction and magnitude of the speaking–writing difference in response timing.

#### 3.2.5. Pragmatic Values, Posterior Policy Beliefs, and Action Probabilities

To characterize how the fitted likelihood asymmetry translated into the expected pragmatic value of alternative response-timing policies, we computed pragmatic values separately for each participant using their fitted CorrS and CorrW parameters. For each policy, the probabilities of correct and incorrect feedback were obtained from the participant-specific fitted likelihood mapping. Because correct feedback was assigned C=0.1 and incorrect feedback C=−0.1, pragmatic value was defined as:

$$V_{prag}\left(\pi \right)=P\left(correct|\pi \right)\left(0.1\right)+P\left(incorrect|\pi \right)\left(-0.1\right).$$

For example, for participant 002, $Corr_{S}=0.601$. Therefore, under the speaking state, the early-response policy implied $P\left(correct\right)=0.601$ and $P\left(incorrect\right)=0.399$, yielding

$$V_{prag}\left(\pi_{early}\right)=\left(0.601\right)\left(0.1\right)+\left(0.399\right)\left(-0.1\right)=0.020.$$

Because the two-parameter model imposes complementarity across response timing, the late-response policy for the same participant implied $P\left(correct\right)=0.399$ and $P\left(incorrect\right)=0.601$, yielding a pragmatic value of −0.020. Thus, pragmatic values were deterministic transformations of each participant’s fitted likelihood parameters under the fixed outcome preferences.

Under speaking, the mean pragmatic value was positive for the early-response policy (M=0.0064) and negative for the late-response policy (M=−0.0064), yielding an average early-over-late pragmatic advantage of 0.0129. In the writing condition, this pattern reversed: the mean pragmatic value was negative for the early-response policy (M=−0.0060) and positive for the late-response policy (M=0.0060), yielding an average late-over-early advantage of 0.0120. Thus, the fitted likelihood mappings implied a modality-dependent pragmatic bias toward early responding under speaking and toward late responding under writing. Because these pragmatic values are deterministic functions of the fitted likelihood parameters and the fixed outcome preferences, they were used primarily to characterize the mechanism through which the likelihood asymmetry enters policy evaluation rather than as an independent inferential outcome.

We next examined whether this theoretically implied asymmetry was expressed in the prospective posterior beliefs over response-timing policies generated by the fitted participant-level models. In the SPM implementation, prospective belief updating over candidate response-timing policies is represented by MDP.R. The mean posterior probability of the early-response policy was 0.503 under speaking (SD=0.004, 95% CI [0.501, 0.505]) and 0.497 under writing (SD=0.004, 95% CI [0.495, 0.499]), yielding a mean within-participant difference of 0.0062. Although this modality-dependent difference in posterior policy belief was small in absolute magnitude, it was consistently expressed across participants. A Bayesian paired-samples t test provided evidence for a modality-dependent difference in MDP.R, $BF_{10}=6.53$. For heuristic characterization of the magnitude and consistency of this difference, the corresponding frequentist paired-samples test yielded $t\left(15\right)=3.052$, p=0.008, mean difference =0.006, 95% confidence interval [0.002, 0.011], and Cohen’s d=0.763, 95% confidence interval [0.194, 1.313]. Thus, the prospective shift in policy belief was numerically small but relatively consistent across participants.

This policy-level asymmetry was more strongly expressed at the subsequent action-selection stage. In the SPM implementation, posterior action probabilities are represented by MDP.P. The mean posterior probability of an early action was 0.551 in the speaking condition (SD=0.063, 95% CI [0.518, 0.584]) and 0.453 in the writing condition (SD=0.064, 95% CI [0.418, 0.487]). A directional Bayesian paired-samples t test provided evidence that $MDP.P_{\mathrm{Early}}$ was greater in the speaking condition than in the writing condition, $BF_{10}=14.15$. For heuristic characterization of the standardized effect, the corresponding frequentist paired-samples test yielded $t\left(15\right)=3.103$, p=0.007, mean difference =0.098, 95% confidence interval [0.031, 0.166], and Cohen’s d=0.776, 95% confidence interval [0.204, 1.328]. Thus, the small modality-dependent asymmetry in prospective policy beliefs was expressed more strongly at the action-selection level.

The resulting action probabilities also closely matched the observed response-timing pattern. The mean posterior probability of an early action was 0.551 in the speaking condition and 0.453 in the writing condition compared with the observed early-response proportions of 0.554 and 0.451, respectively. Thus, the POMDP translated a relatively subtle modality-dependent difference in prospective policy belief into a larger action-level difference that closely reproduced overt behavior.

However, this correspondence alone does not establish that the additional latent structure of the POMDP is warranted. A simpler behavioral model could potentially reproduce the same early-versus-late response distribution without representing modality-specific likelihood mappings, pragmatic policy evaluation, posterior beliefs over policies, or their transformation into action probabilities. We therefore asked whether the additional inferential structure embodied by the POMDP provided sufficient explanatory value to justify its greater complexity. To address this question, we compared the POMDP with the simpler logistic-link VL model using Bayesian model selection.

#### 3.2.6. Model Comparison with the Logistic-Link VL Model

We compared the POMDP with the simpler logistic-link VL model fitted to the same participant-specific median-discretized response-timing outcome. The logistic-link model provided a behavioral benchmark because it estimated the association of production modality and accuracy with early-versus-late responding (Table 2) without representing the latent inferential sequence embodied by the POMDP. In contrast, the POMDP additionally represented modality-dependent feedback likelihoods, prospective posterior beliefs over response-timing policies (MDP.R), and the transformation of those beliefs into posterior action probabilities (MDP.P).

We compared participant-specific model evidence using random-effects Bayesian model selection. The comparison favored the POMDP, yielding a protected exceedance probability of 0.99 and a Bayesian omnibus risk of less than 0.001. Thus, within the present comparison set, the increase in model evidence associated with the POMDP was sufficient to offset the complexity introduced by its additional latent computational structure.

## 4. Discussion

The present study developed a formal active-inference account of the speaking-over-writing advantage in the retrieval of the production modality of individual words. At the behavioral level, participants identified the production modality of spoken words earlier and more accurately than that of written words. These behavioral findings stand independently of the computational interpretation developed here. The POMDP provides one formal account of this pattern by representing speaking and writing as contextual states under which alternative response-timing states differ in their expected feedback consequences. Within this model, the behavioral asymmetry was expressed as a production-modality-specific difference in the likelihood mapping from response timing to feedback: the early-response state was more strongly associated with correct feedback in the speaking condition than in the writing condition.

The principal computational result was the group-level difference between the CorrS and CorrW parameters. CorrS quantified the model-implied likelihood of correct feedback given the joint configuration of the early-response and speaking states, whereas CorrW quantified the corresponding likelihood under the early-response and writing states. The posterior contrast indicated that CorrS was greater than CorrW.

In the POMDP architecture, production modality was not modeled as a state that the agent had to discover during each trial. Instead, speaking or writing provided the contextual state under which the expected consequences of early- and late-response policies were evaluated. A selected policy moved the agent into an early- or late-response state, and the joint configuration of the response-timing state and the production-modality state determined the expected feedback observation through the likelihood mapping. Thus, the model provides a formal mechanism linking production context, response timing, and expected feedback.

Because correct feedback was preferred and incorrect feedback was avoided, differences in these likelihood mappings produced differences in the expected pragmatic value of the available response-timing policies. When CorrS > 0.5, the joint configuration of the early-response and speaking states is more likely than the corresponding late-response configuration to generate correct feedback. Conversely, when CorrW > 0.5, the late-response state under writing is more likely than the early-response state to generate correct feedback. Within the model, these likelihood differences therefore produce a pragmatic bias toward relatively early responding under speaking and relatively late responding under writing, which is subsequently expressed in posterior policy beliefs and action probabilities.

This formulation specifies the role assigned to sensory consequences within the present account. In the real-life example introduced at the beginning of the paper, the correctness of a retrieved name is not directly observable before acting. What can subsequently become observable is the consequence of saying the name, such as a smooth continuation of the interaction, confusion, or correction. The expected response of the other person can therefore contribute prospectively to the evaluation of whether and when to act on the retrieved name. This does not imply that social approval is sought independently of retrieval accuracy; rather, such feedback is relevant insofar as it is expected to follow a correct or incorrect response.

The findings also speak to active-inference accounts in which precision modulates the influence of sensory evidence on beliefs (Feldman & Friston, 2010; Parr & Friston, 2017, 2019; Parr et al., 2022). Specifically, more reliable likelihood mappings produce more differentiated beliefs about the consequences of alternative policies. In the context of the present task, the estimated mappings suggest that the expected consequences of early and late responding differed more clearly under the speaking state than under the writing state. This interpretation concerns precision within the formal model and should not be taken as direct evidence of attentional gain, neural precision, or a particular physiological mechanism.

We consider it important to note that the present results do not establish this feedback-related likelihood mechanism as a unique explanation of the speaking-over-writing advantage. Other accounts—for example, differences in evidence accumulation, source-memory strength, contextual reinstatement, or other retrieval processes—could potentially generate the same behavioral pattern. The contribution of the present POMDP is therefore more specific: it shows that the observed speaking-over-writing difference can be represented within an explicit generative architecture in which modality-dependent likelihood mappings produce differences in expected pragmatic value, prospective policy beliefs, and ultimately, response-timing probabilities. Distinguishing this account from alternative computational mechanisms will require direct model comparisons in future work.

### 4.1. Relation to the Comparison Model

A comparison logistic VL model and the POMDP were fitted to the same behavioral outcome: whether a response occurred relatively early or late. The logistic VL model only provided behavioral account by estimating early-versus-late responding as a function of production modality and response accuracy. In contrast, the POMDP addressed the manuscript’s primary theoretical question by representing a generative sequence in which production modality contextualized the likelihood mapping between response-timing states and subsequent feedback. These likelihood mappings, together with outcome preferences, determined the expected pragmatic value of alternative response-timing policies and contributed prospectively to posterior policy beliefs and action probabilities. Crucially, the model comparison answered the complementary question of whether introducing this latent structure is supported relative to a simpler account of the same early-versus-late behavioral outcome.

### 4.2. Scope of the Interpretation in the Context of Discourse Production

The present task examined source recognition for individually produced words. It was not designed to address the broader cognitive or epistemic functions of speaking and writing (Limongi et al., 2024; Silva & Limongi, 2017, 2019). Writing is typically slower than speaking and may support greater elaboration, externalization, revision, and development of knowledge (Baaijen & Galbraith, 2018; Biber, 1988). These properties may be advantageous in tasks involving sentence production, conceptual organization, learning, or long-term knowledge construction, even though writing was associated here with slower and less accurate recognition of the production modality of individual words.

The speaking-over-writing advantage observed in this task should therefore not be interpreted as a general cognitive superiority of speaking over writing at the discourse level. It is specific to the retrieval of how isolated words were produced under the present experimental conditions. Future studies could examine whether the same parameter pattern is observed when participants produce sentences, extended discourse, or semantically integrated material. Such tasks may reveal different relations among production modality, response timing, and expected outcomes.

The present modeling approach could also be adapted to the broader recognition advantage observed when produced items are compared with silently read items, often termed the production effect (MacLeod & Bodner, 2017; MacLeod et al., 2010). Such an extension would require a task and model architecture different from those examined here. The present model concerns source judgments between speaking and writing and represents production modality as a contextual state that constrains the expected consequences of early and late responding. A produced-versus-silent recognition model would instead need to represent whether an item had been produced, how production-related contextual features distinguish studied items from lures, and how those features contribute to old–new recognition judgments. The present findings therefore do not directly explain this broader phenomenon. Rather, the model provides a formal element—context-dependent mappings among retrieved features, response policies, and feedback—that could be examined in a task designed specifically for that purpose.

Extending the model in this direction could also permit a constructive comparison with established computational accounts of recognition memory. Global-matching models such as MINERVA 2 (Jamieson et al., 2016) and REM (Kelly et al., 2022) formalize recognition in terms of the similarity between retrieval cues and stored traces, whereas distinctiveness-based accounts emphasize the diagnostic value of features generated during production. An active-inference formulation could complement these approaches by representing how retrieved contextual evidence informs action selection in relation to expected sensory consequences. Future work could examine whether these frameworks provide compatible, partially overlapping, or distinct descriptions of produced-versus-read recognition when evaluated using comparable behavioral data. The present study should therefore be viewed as a restricted foundation for such future comparisons, rather than as a model of the production effect itself.

### 4.3. Limitations to Be Addressed in Future Studies

While the present results open a new research avenue in the area of language production and active inference, several limitations need to be addressed in future studies.

First, based on the participant homogeneity and sample size, the robustness and generalizability of the results are limited to comparable participants and comparable experimental conditions.

Second, continuous response times were represented as early and late response policies. This distinction was introduced to instantiate alternative temporal policies within the discrete POMDP architecture, rather than simply as a convenient statistical reduction. Nevertheless, assigning responses to two policy classes necessarily omits variation within each class. Future work should compare the present formulation with models that retain continuous response times or represent a greater number of temporally extended policies.

Third, the model contained only two timesteps and two response-timing policies. This intentionally minimal architecture captures the decision to act relatively early or to delay responding, but it does not specify the processes occurring during the delay. A later response could reflect uncertainty, hesitation, additional retrieval operations, motor preparation, or other processes. A richer model could include additional timesteps and an explicit wait policy, allowing the consequences of continued sampling or delayed commitment to be examined without assuming that later responses necessarily reflect evidence accumulation.

Fourth, feedback was represented within each trial as an observation generated by the joint configuration of production modality and response timing. In the experimental task, however, feedback may also have influenced expectations across trials. The current model does not estimate such learning. A hierarchical or temporally extended model could allow participants to update beliefs about the reliability of early and late responding over the course of the experiment.

Fifth, the production-modality state was fixed by the trial structure. The model therefore characterizes how the speaking or writing context changes expected policy outcomes, but it does not model the initial reconstruction or inference of production modality itself. A more complete source-memory model could represent uncertainty over whether the probe had been spoken or written and examine how modality-specific contextual evidence contributes to resolving that uncertainty.

Sixth, the model did not separately represent recognition accuracy and response timing as distinct outcome modalities. Instead, feedback was generated from their modeled relationship under the production-modality state. Although this architecture served the present theoretical objective, alternative formulations could model the recognition choice, response latency, confidence, and feedback as separate but interacting observations.

Finally, the model was compared with a relatively simple linear alternative. The model-selection result therefore provides evidence only within the tested comparison set. Future work should compare the present POMDP with other plausible generative accounts, including global-matching models, source-memory models, sequential-sampling models, and active-inference models with continuous or more deeply structured temporal policies.

## 5. Conclusions

The present study provides a preliminary formal account of the speaking-over-writing advantage in production-modality retrieval. Behaviorally, participants identified spoken productions more quickly and accurately than written productions. Within the POMDP, this advantage was expressed as a stronger association between early responding and correct feedback under the speaking state than under the writing state. The positive (CorrS-CorrW) contrast therefore constitutes the primary computational signature of the empirical pattern within the present model.

By representing production modality as a contextual state, response timing as policy selection, and correct or incorrect feedback as an observable sensory consequence, the model connects the behavioral pattern to an explicit inferential architecture. The results do not establish this architecture as a complete or unique explanation of the speaking-over-writing advantage. Rather, they show that an intentionally minimal active-inference model can formalize how spoken and written production contexts may differentially constrain the expected consequences of acting relatively early or late on a recognition belief.

This framework provides a basis for further comparison with alternative models and for extensions that represent uncertainty about production modality, continuous response timing, learning across trials, and richer forms of linguistic production. In this sense, the present contribution is a focused first step toward modeling how production-modality context and expected sensory consequences jointly contribute to recognition behavior.

## Figures and Tables

**Table 1:** Descriptive statistics.

	Speaking (M ± SD)	Writing (M ± SD)
Number of Trials	82.50 ± 8.76	82.94 ± 8.07
RT (s)	1.64 ± 0.38	1.77 ± 0.40
Proportion Correct	0.88 ± 0.07	0.81 ± 0.12
Proportion Incorrect	0.12 ± 0.07	0.19 ± 0.12

Note. Values represent subject-level means (M) and standard deviations (SD).

**Table 2:** Descriptive statistical models’ parameter estimates and uncertainties.

Model	Outcome	Variable	Level/Contrast	Mean	SD	95% Credible Interval	
						Lower	Upper
ANCOVA	Response Time (Continuous)	Intercept		1.704	0.106	1.491	1.912
		Production Modality	Speaking	−0.064	0.013	−0.091	−0.04
			Writing	0.064	0.013	0.036	0.088
	Accuracy	Intercept		0.843	0.033	0.776	0.907
		Production Modality	Speaking	0.032	0.007	0.018	0.046
			Writing	−0.032	0.007	−0.048	−0.02
Logistic VL-PEB	Response Time (Discretized)	Production Modality	Writing vs. Speaking	0.373	0.101	0.176	0.571

**Table 3:** Subject-level likelihood parameter estimates.

Participant	CorrS	CorrW
001	0.492	0.511
002	0.601	0.396
003	0.541	0.462
004	0.540	0.465
005	0.546	0.459
006	0.521	0.479
007	0.536	0.466
008	0.521	0.479
009	0.551	0.450
010	0.559	0.445
011	0.536	0.466
012	0.411	0.594
013	0.514	0.490
014	0.535	0.469
015	0.548	0.454
016	0.561	0.439

## Data Availability

Modeling scripts and raw data files can be downloaded from the Writing-brain laboratory website https://people.brandonu.ca/limongir/home/the-writing-brain-laboratory/ (accessed on 18 July 2026).
